# A Comparison of Dietary Patterns and Factors Influencing Food Choice among Ethnic Groups Living in One Locality: A Systematic Review

**DOI:** 10.3390/nu14050941

**Published:** 2022-02-23

**Authors:** Grace Bennett, Laura A. Bardon, Eileen R. Gibney

**Affiliations:** 1 Institute of Food and Health, University College Dublin, Belfield, D04 V1W8 Dublin, Ireland; grace.bennett@ucdconnect.ie (G.B.); laura.bardon@ucd.ie (L.A.B.); 2 School of Agriculture, Food Science and Veterinary Medicine, University College Dublin, Belfield, D04 V1W8 Dublin, Ireland

**Keywords:** ethnicities, minority groups, ethnic diets, dietary comparisons, influencers of food choice

## Abstract

Globally, the number of minority ethnic groups in high-income countries is increasing. However, despite this demographic change, most national food consumption surveys are not representative of ethnically diverse populations. In consequence, many ethnic minorities’ dietary intakes are underreported, meaning that accurate analysis of food intake and nutrient status among these groups is not possible. This systematic review aims to address these gaps and understand differences in dietary intakes and influencers of dietary habits of ethnic groups worldwide. A systematic search was conducted through three databases (Pubmed, Web of Science and Scopus) and manual searches, generating *n* = 56,647 results. A final search of these databases was completed on 13 September 2021, resulting in a total of 49 studies being included in this review. Overall, food group intakes—particularly fruit, vegetable and fish intake—and diet quality scores were seen to differ between ethnicities. Overall Black/African American groups were reported to be among the poorest consumers of fruit and vegetables, whilst Asian groups achieved high diet quality scores due to higher fish intakes and lower fat intakes compared to other groups. Limited data investigated how nutrient intakes, dietary and meal patterns compared between groups, meaning that not all aspects of dietary intake could be compared. Socioeconomic status and food availability appeared to be associated with food choice of ethnic groups, however, confounding factors should be considered more closely. Future work should focus on comparing nutrient intakes and meal patterns between ethnicities and investigate potential targeted interventions which may support adherence to food-based dietary guidelines by all ethnic groups.

## 1. Introduction

The rate of ethnic diversity in developed countries is continuously increasing. In the United States (US) nearly 40% of the population are non-White, with Hispanic/Latino (18.5%) and Black/African American (13.4%) the largest ethnic minorities in the US [1]. Although only 16% of adults in the European Union were foreign nationals in 2013, this trend is on the rise with nearly one quarter of young European adults now having a migrant background [2]. Integration can be challenging for immigrants in terms of adapting to new lifestyles, cultures and dietary norms. While many studies have examined dietary acculturation of individual ethnic minorities post migration [3,4,5,6], limited research has comprehensively collated data comparing the dietary habits of different ethnic groups globally, nor has it examined what drives certain food practices of different ethnicities. There is also a paucity of research comparing multiple aspects of dietary intake among ethnic groups as variances in dietary data collection and analysis hinder comparison of nutritional data [7].

Knowledge of dietary differences is important to help identify specific ethnicities at risk of nutrient inadequacies, or which groups may need additional support in reaching food group intake recommendations. Minority groups residing in high-income countries are known to be more at risk of developing non-communicable diseases with some of this disparity believed to be attributable to modifiable risk factors, including dietary intake [8,9,10]. As such, it is critical that ethnic minorities are provided with ample guidance and opportunities to adhere to healthy eating recommendations, especially in anticipation of a global shift towards more sustainable, plant-based diets [11]. Once dietary disparities between ethnicities are identified, it is imperative to understand the factors which influence food choice among groups. Without knowledge of the factors that impact dietary habits, effectively addressing inadequacies among population groups and adapting public health campaigns accordingly would prove difficult. Previously, “social and cultural environment” and “food beliefs and perceptions” were reported as common influencers of food choice amongst almost all minority groups across Europe [12]. However, this research noted that the range of factors influencing food choice is broad, and direct comparison between population groups is lacking [12].

Reliable in-depth analysis of dietary habits of different population groups is required if future food-based recommendations can be tailored to specific ethnicities. Furthermore, when implementing effective health campaign strategies, it is crucial to understand reasons for certain dietary habits among groups. With this knowledge, health campaigns can be tailored, not only to groups at risk, but also to factors influencing what they eat. This systematic review aims to identify key dietary trends of ethnic groups globally and examine the factors that affect food choice. By contributing to the understanding of dietary differences, this research aims to inform where aspects of public health policy and guidelines need to be developed to cater for multi-ethnic populations. 

## 2. Materials and Methods

This systematic review was conducted from October 2020–November 2021 in line with the updated Preferred Reporting Items for Systematic Reviews and Meta-analysis (PRISMA 2020) guidelines [13]. The protocol of this review has been registered on PROSPERO’s international database (registration number: CRD42021231409).

### 2.1. Search Strategy and Eligibility Criteria

A comprehensive search strategy, following the PICO framework, was utilised (Supplementary Table S1). The search strategy focused specifically on two research questions: (1) how dietary patterns differ between ethnic groups in one locality, and (2) what factors influence food choice of ethnic groups. A consensus from all researchers was reached on the following search terms: ((ethnic* OR race OR immigrant* OR minorit* OR Asia* OR China OR Chinese OR Africa* OR Black OR “Non-Hispanic” OR Hispanic OR Latino OR Brazil* OR “Eastern Europe*” OR Polish OR Poland) AND (“diet* pattern” OR “diet* intake” OR “food intake” OR “food consumption” OR “food group”) AND (factor OR influence OR “food choice” OR reason OR determinant) NOT (child*)). Two researchers independently performed database searches using Pubmed, Scopus and Web of Science between 11 October 2020 and 21 December 2020. The final search of all databases was completed by one researcher on 13 September 2021.

Studies that considered at least two ethnicities living in one country and provided a clear comparison of dietary habits or influences of food choice between ethnicities were considered. Only papers that involved adult cohorts were included. Primary research studies, papers published in all languages, and in all years, were included in the eligibility criteria. Papers published in languages other than English were only excluded during abstract or full-text screening if there was no English version available from other sources. Published papers were only excluded based on year if there was a more recent study, of the same research design, available since publication. Research involving child or adolescent cohorts, pregnant or breastfeeding women and/or disease cohorts were excluded. Papers that examined association of diet with disease, did not provide sufficient information on overall diet, or those which focused on dietary acculturation within one ethnic group, were not included. Review papers or opinion articles, including systematic and narrative reviews or commentaries, were excluded. Reference lists of all reviews found in the primary search were checked, and any relevant papers were added to the list of total studies to be screened (*n* = 73).

### 2.2. Study Screening and Quality Assessment

Two researchers independently screened all papers based on title, followed by abstract, and lastly by full text [14]. Search results from all three databases were imported into EndNote X9 where all duplicate titles were removed. Once irrelevant titles were removed, the remaining studies were uploaded to Covidence, where studies were screened based on abstract and full text against the inclusion and exclusion criteria [15]. When the two researchers’ final lists of included studies were retrieved, all researchers came together to discuss any discrepancies between the two study lists. The “Quality Assessment Tool for Cohort and Cross-Sectional Studies” was used to assess the quality of all included quantitative studies. To assess bias, quantitative studies were scored based on 14 questions and classified as “Good”, “Fair” or “Poor” quality. The quality of qualitative studies (*n* = 3) was not examined [16,17,18].

### 2.3. Data Extraction and Synthesis

Method of dietary assessment and presentation of dietary analysis (e.g., food group and nutrient intakes, dietary patterns and dietary quality) varied between studies reported. To allow for easier aggregation of findings, papers were collated based on method of analysis. Intakes from specific food groups were presented as mean daily servings where possible (*n* = 6 studies). Where papers reported intakes from food groups as grams per day (g/day), mean daily servings of each food group consumed were calculated from appropriate recommended serving sizes, indicated in the relevant tables [19]. As a result, any statistical significance originally reported in these studies does not apply to the revised data. Intake from food groups was also reported as percentage consumers in some papers (*n* = 5 studies [20,21,22,23,24], Supplementary Table S4). As diet quality was assessed through multiple dietary indices a narrative summary of scores and key drivers of quality is provided.. A narrative summary of findings on meal patterns of groups is provided, as data presentation varied considerably between studies and did not allow for quantitative analysis (*n* = 5 studies [25,26,27,28,29], Supplementary Table S5). Similarly, methods used to assess the factors influencing food choice among ethnicities varied greatly, with studies using regression and clustering analyses, and qualitative methods. Data on the factors which influence food choice are presented under food availability, socio-economic status (SES) and health concerns. When classifying ethnicity, international reporting guidelines were considered [30,31,32]. The following terms will be used to report ethnicity throughout this review, regardless of the terminology used by individual studies: Asian, Black, Hispanic, Latino, Native Hawaiian, Pacific Islander and White. The only exception is when papers list country specific ethnicities (e.g., Chinese, Mexican, Nigerian).

## 3. Results

**Figure 1:**
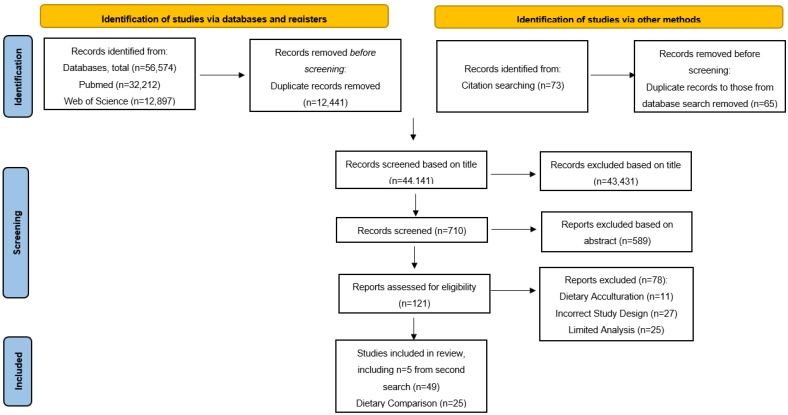
PRISMA Flow Diagram.

The search strategy produced a total of 56,647 results from database (*n* = 56,574) and manual searches (*n* = 73). Following the removal of duplicates, *n* = 43,431 studies were excluded based on title, *n* = 589 were excluded based on abstract and *n* = 78 studies were excluded based on full text (Figure 1). Following a second database search, a total of 49 studies were included after full text articles were screened against the eligibility criteria. Of these studies *n* = 25 looked at diet only, *n* = 11 examined influences of food choice only and *n* = 13 examined both.

### 3.1. Detail of Included Studies

An overview of each study included in this review is summarised in Supplementary Table S2 (studies examining dietary habits) and Supplementary Table S3 (studies reporting food choice influences). Nearly all (*n* = 46) studies were quantitative in design, most of which were cross-sectional studies (*n* = 44). Of the papers that examined diets between ethnicities, *n* = 24 used a food-frequency questionnaire (FFQ), *n* = 10 used 24-h dietary recall (24 HDR) and *n* = 2 used a diet history or questionnaire. Two papers used a combination of these methods [33]. Data on influences of food choice were collected via questionnaires (*n* = 15), census analysis (*n* = 5) and structured focus groups (*n* = 4). Studies were primarily conducted in developed countries, but ranged globally, spanning North America (*n* = 31), Europe (*n* = 8), Asia (*n* = 3), Africa (*n* = 4) and Oceania (*n* = 3). In terms of quality assessment, *n* = 26 studies were classified as “Good” quality, with *n* = 20 classified as “Fair” quality. No study was rated as “Poor” quality. Many studies classified as “Fair” did not state an eligibility rate of at least 50% or provide justification for the chosen sample size.

### 3.2. Dietary Intake Comparisons among Ethnicities

Dietary comparisons among different ethnicities were examined by *n* = 38 studies (Supplementary Table S2). These comparisons included evaluation of food group intakes, macro and micronutrient intakes, dietary and meal patterns and diet quality.

#### 3.2.1. Food Group Intake

A total of *n* = 30 studies assessed and reported food group intakes. Studies where food group intakes could be presented as mean servings per day are reported in Table 1. 

Fruit and Vegetables 

Black groups in the US reported consuming significantly fewer vegetables compared to White and Hispanic groups, while Hispanic and Latino groups had higher fruit intakes than White groups [20,21,22,23,34,35,36,37] (Table 1 and Supplementary Table S4). Black groups were also least likely to meet recommended vegetable intakes (47%) compared to Latino (52–65%) and White groups (63%) in the US [35]. Findings among Asian groups were more inconsistent where South Asian Surinamese in the Netherlands consumed fruit and vegetables more frequently than their Dutch counterparts [22]; however, in the US and Australia, Asian and Pacific Islanders consumed fruit and vegetables less frequently than White groups [35,38,39]. Adebayo et al., 2017, reported the lowest daily consumers of fruit and vegetables overall, whereby nearly no Somalians in Finland reported consuming fruit and vegetables at least six days weekly, compared to over 50% of Russian and Kurdish groups [24] (Supplementary Table S4).

Protein Foods—Meat and Eggs

Several studies reported low red meat intake among Asian and Black groups compared to White and Hispanic groups [22,39,40] (Table 1). The White group were reported to have lower servings of chicken compared to other groups, but statistical significance was not assessed [34,41]. Fish was the food group with the lowest number of mean daily servings regardless of ethnic group. Asian groups consistently had the highest fish intake, except for Pacific Islanders in New Zealand (0.43 vs. 0.63 servings/day) [22,39,41]. Fish equated to ~15% of Native Hawaiian and Asian groups’ total protein intake, compared to 6% of Black and 3% of Latino [40]. Elsewhere, in Finland, half as many Kurdish participants reported consuming fish more than twice weekly than Somalian and Russian groups (21% vs. 41% and 43%, respectively), Supplementary Table S4 [24]. 

Dairy

All studies reported that White groups had the highest servings of milk and cheese per day (up to 2.17 servings daily) compared to Black (0.58 servings daily, US), Asian (1.3 servings daily, Australia), Maori (0.3 servings daily, New Zealand) and Pacific Islander (0.1 servings daily, New Zealand) groups [34,39,41,42] (Table 1). However, similar percentages of White (77.3%) and Hispanic (76.8%) groups in the US were milk consumers, with both groups reporting one serving daily [34] (Supplementary Table S4). Based on contribution to diet quality scores, Black males in the US had significantly lower dairy intake than their White counterparts [43].

Snacks and Fast Food

“Unhealthy” foods were broadly defined as snacks, desserts and soft drinks. Recently in the US, the Black group were reported to be higher consumers of “unhealthy” foods than the White group (72.8% vs. 62.2%) [20] (Supplementary Table S4) and consumed significantly more servings of snacks and desserts (2.4 vs. 2.1 servings/day) [42] (Table 1). Black groups living in the US were more frequent consumers of fast-food than White groups [34,42] (Supplementary Table S4). In the US, soft drinks were the top contributors to added sugar intake [44].

#### 3.2.2. Nutrient Intake

Six studies examined mean daily energy intake, but not all reported standardising intakes to body weight or recommended intakes, thus making them difficult to compare and interpret [33,39,43,45,46,47] (Table 2). Many ethnic groups in the US and Oceania consumed well in excess of their protein requirements [39,41,43,46]; however, the Kamba ethnic group in Kenya had significantly lower mean daily protein intakes than any other group (male: 49.60 g/day, female: 38.50 g/day) [47]. Rice was the top protein contributor for the Asian group (12%), where chicken was the main dietary source of protein among Black, White, Native Hawaiian and Latino groups in the US [44]. In terms of total fat intake, again the Kamba group had significantly lower mean intakes than other ethnicities in Kenya [47]. Asian groups in Oceania also had significantly lower total fat intakes than White, Pacific and Maori groups [39,41]. Carbohydrate intake was lowest among Asian groups compared to other groups in Australia, New Zealand and the US [39,41,45]. Minimal differences in carbohydrate intake between American White and Black groups were noted [43,46]. Considering the type of carbohydrate, Asian groups in New Zealand and the US had significantly lower sucrose intakes than other groups assessed (e.g., Chinese: 62.2 g/day vs. Nigerian: 147.6 g/day and Mexican: 130.1 g/day) [41,45], and the Arab group in Tunisia had significantly higher sucrose intakes than the Berbe group (33 g/day vs. 23 g/day) [33]. In the US, less than 5% of White and Black males met total sugar recommendations [43]. Black groups in the US had higher daily mean intakes of dietary fibre than Asian groups, but lower than Hispanic and White groups, with 2.1% of Blacks meeting recommendations [41,43,45]. Bread and cereals contributed most to fibre intakes (18–22%), however, beans were also an important fibre source for the Latino group [44].

#### 3.2.3. Diet Quality and Dietary Patterns

Dutch and African Surinamese groups living in the Netherlands had significantly lower diet quality scores (based on the Dutch Healthy Eating Index (DHEI), 2015) [48] than their Asian Surinamese, Turkish and Moroccan counterparts [22,49] (Table 3). Examining drivers of diet quality, Dutch males had the lowest vegetable intake, with both Dutch males and females consuming the most processed/red meat [22,49]. African Surinamese had the highest intake of sugar sweetened beverages and fruit juices while Asian Surinamese were the highest fish consumers of all groups (>80% consuming fish at least once weekly) [22,49]. Although no significant differences in overall Healthy Eating Index (HEI) scores [50] between White and Black males and females in the US were reported, drivers of diet quality scores did differ, particularly among males [43,46]. Black males had significantly lower scores for fruit, vegetables, dairy, seafood and plant protein than White males in the US [43]. In Australia, Asian groups were found to have healthier eating behaviour scores than other groups assessed [51]. Again, high fruit and vegetable intake and low fast food and processed meat consumption contributed to higher eating scores [51] (Table 3). 

Five studies compared dietary patterns among ethnic groups [25,26,27,28,29] (Supplementary Table S5). Dietary patterns were categorised differently across studies; however, three studies identified distinct trends of fruit, vegetables and nuts (e.g., “Prudent pattern”), red meat, sweets and snacks (e.g., “Western pattern”) and white meat, refined grains and legumes (e.g., “Eastern pattern”) [26,27,29]. Dietary patterns were found to differ significantly between ethnic groups, with Asian groups being more likely to adhere to the Eastern dietary pattern than White groups who were more likely to adhere to the Prudent dietary pattern [26,27]. The Western pattern was associated with lower fibre, vitamin C and non-haem iron intakes, while the Prudent dietary pattern was associated with higher intakes of these nutrients [27]. 

### 3.3. Food Choice Influences

A total of *n* = 24 studies examined factors influencing food choice among ethnic groups (Supplementary Table S3). Studies identified multiple determinants of food choice through various methods of analysis (Table 4). Findings are presented below under three main headings of SES, food price and availability, and health concerns.

#### 3.3.1. SES: Education and Occupation

The influence of SES on diet was stratified by three to four SES levels across each ethnic group assessed in the Netherlands, US and Iran [22,29,49,52]. High education levels were positively associated with fruit and vegetable consumption among Afro-Caribbean and Asian Surinamese in the Netherlands, Turkish and Kurdish in Iran and White groups in the US [22,29,52] (Table 4). Education was positively associated with adherence to healthier dietary patterns among White and Black groups; this association was significantly stronger among the White group [53]. Occupation level among Dutch and Moroccan groups, and Surinamese females in the Netherlands and Turkish and Kurdish groups, was also associated with diet quality scores and dietary patterns [27,29,49] (Table 4). 

#### 3.3.2. Food Price and Availability

The influence of price and convenience differed across studies, with food price and convenience found to be less important to White than Hispanic and Black groups in the US [54] (Table 4). However, food price was ranked significantly higher by White students compared to Asian students living in the US [55]. With respect to availability, studies were limited (*n* = 6) and all were conducted in the US, making them difficult to consider in a wider global context. Supermarkets were most common in predominately White areas and no supermarket was found in predominately Black communities [56,57]. In addition, predominantly Hispanic and Asian neighbourhoods also had about one third fewer supermarkets than NH White neighbourhoods [58] (Table 4). Although neighbourhood SES influenced the availability of supermarkets in White and racially mixed areas (with low-income communities less likely to have shops nearby), store availability in predominately Black neighbourhoods was minimal, regardless of SES level [56]. Black and Hispanic groups discussed the lack of supermarkets nearby and highlighted the difficulty of accessing fresh produce or familiar traditional foods in their local grocery stores [17]. Availability of fresh produce was most common in White areas compared to stores in racially mixed and Black areas (64% vs. 31% and 5%, respectively) [57] (Table 4). Availability of fresh fruit and convenience stores was associated with fruit, fat and soft drink consumption among White groups only [57,59], while ethnic food store availability was positively associated with Hispanic fruit and vegetable intake [52].

#### 3.3.3. Health Concerns

In the US, Black, Hispanic and White groups reported the importance of daily fruit and vegetable consumption and limiting fat, sugar and salt intake where fast food was universally seen as unhealthy [17] (Table 4). Awareness of health benefits of certain foods was positively associated with higher diet quality scores, fruit and vegetable intake and lower unhealthy food intake [20]. In the US, the White group reported significantly better knowledge of health risks associated with diet than the Black group [20]. SES was a bigger influence on dietary intake among Black and Hispanic groups than health and nutrition awareness [54]. Asian and Hispanic groups were conscious of artificial additives in food [18,55]. Asian college students believed natural content and ethical considerations were more important than their White counterparts (Chinese: 2.66 and 1.88, American: 1.75 and 1.75) [55] (Table 4). 

## 4. Discussion

Food consumption and dietary intake patterns of ethnic minorities are often underrepresented in national food consumption surveys [8]. There is a need to fully understand dietary differences between various ethnicities and drivers of food choice among groups, as knowledge of disparities among population groups can help inform dietary guidelines specific for ethnicities and develop specifically tailored interventions to effectively encourage dietary change in the long-term [60,61]. This review aimed to compare reported dietary intakes of different ethnic groups and understand possible facilitators and barriers to food choice among different ethnicities.

Differences in dietary intakes and the factors influencing food choice were examined in papers considered in this review, where some consistent findings were seen. Black groups were reported to have among the lowest fruit and vegetable intakes and are reported here and elsewhere not to meet daily recommendations [20,34,35,37,42,43,62,63]. Hispanic and Latino groups had higher fruit and vegetable intake than other ethnicities, however, a large proportion were also reported not to meet recommendations [35,63,64]. This suggests that, despite variation in intake between groups, overall fruit and vegetable intakes are low across the global population, and targeted public health campaigns continue to be required to promote better adherence to healthy eating guidelines [65,66,67]. Another consistency throughout studies was that fish is not frequently consumed by many groups, with White and Hispanic groups amongst the lowest consumers, suggesting that White and Hispanic groups, who are also traditionally high consumers of red meat, may benefit from specific consideration to promote fish consumption. This is even more important currently, as a shift towards sustainable eating patterns is imminent [68,69,70]. 

Whilst some key findings could be elucidated from this work, this review also highlighted the lack of data comparing nutrient intakes across multiple ethnicities. Knowledge of nutrient intakes across ethnicities is necessary to ensure nutrient requirements of all ethnic groups can be met within current food-based guidelines, and when promoting a transition to more sustainable or plant-based diets [71]. With recommendations for reductions in total protein and consumption of more plant-based protein sources, it will be important to determine if, and how, sustainable eating guidelines impact each population group individually [11,72]. To ensure adequate nutritional intake across ethnic groups there is a clear need for ethnic specific dietary guidelines [73,74]. Food-based dietary guideline recommendations, accommodate choice for those with restricted diets, such as vegetarians and vegans [73,75,76], and are developed for age specific groups, e.g., older adults, to tackle nutritional issues associated with ageing [77]. One could argue that a similar approach should be taken for minorities whose nutritional intake and guidance requirements differs to that of the general population. Moreover, as countries develop sustainable food-based dietary guidelines, different ethnicities should be considered separately, as food group intake in this review is shown to differ among groups. Research into how meal patterns compare among ethnicities is also limited. Meal pattern analysis provides a unique insight into dietary habits, as it examines diet overall, taking all components (food and nutrient consumption) into consideration, and is often used when considering the relationship between nutrition and disease [78]. Minority groups are often reported to be at higher risk of developing chronic diseases, such as diabetes, cancer and cardiovascular disease, as well as of delayed diagnosis or treatment for such diseases [8,79,80], the risks of which are known to be influenced by diet and lifestyle [8,10]. However, it is difficult to ascertain the true association of diet with disease development among ethnic minorities as national dietary surveys do not provide sufficient data on ethnic groups to facilitate in-depth analysis [8]. 

In addition to understanding what people are eating, it is also important to understand the underlying reasons why, and this was also examined within this review. SES was reported to be an influential factor in ten of the studies considered in this review [22,23,27,29,49,52,53,54,81,82]. Previously, education and income have been significantly associated with healthier and more diverse food intake, including higher fruit, vegetable and fish consumption [83,84,85,86]. Yet when examining the association of SES with diet in this review, it was difficult to interpret, due to the variation in confounders used to control for other social and demographic factors, an important issue that limits how results can be compared [87]. Detailed and comprehensive research into the impact of SES across population groups, particularly ethnic groups, is warranted. Understandably, food choice did depend on food availability and shop proximity; however, this was only assessed in the US [17,23,56,57], and a more global approach is required to assess the true impact of food availability on the diet diversity of different ethnicities. Whilst results found in this review suggest SES and food availability are key drivers of food choice, limited data across ethnicities means there is little evidence to drive targeted public health campaigns. Interventions that involve multiple factors (including nutrition counselling and physical activity), and are family orientated, prove more effective among minorities [88]. More research into the effectiveness of educational interventions among adults of ethnic minorities needs to be undertaken to encourage the introduction and success of such campaigns globally [89,90,91,92].

Finally, the assessment of dietary intake and food choice among minority groups is challenging due to a range of factors, including inappropriate assessment methods, which vary from one study to another, as well as variation in recruitment approaches and cultural engagement across ethnic groups [93]. One way around this is to consider how existing data arising from different studies can be compared to consider differences in intake across groups. Using this approach more local and diverse studies can be used to answer this single research question. Adopting a standardised approach to the collection of food intake and food choice data could support exchange of validated databases and lead to more in-depth comparison across a myriad of ethnicities globally. This challenge is currently being addressed in projects such as the Food and Nutrition Security-Cloud (FNS-Cloud) [94] and the European Science Open Cloud (EOSC) [95]. 

Although this systematic review provides comparisons of dietary intake between host and migrant populations, limited conclusions can be drawn from these findings. Dietary intake was primarily described as food group intake by studies included in this review, so nutritional status and dietary patterns of multiple ethnicities could not be fully compared. Data depositories which facilitate exchange of expansive, standardised and validated data would support more in-depth comparisons of similar dietary and food choice data [94]. In addition, not all studies adjusted for confounding factors, so conclusions must be interpreted with caution. Few studies included in this review achieved a similar distribution of ethnicities to facilitate in-depth comparisons. As there is no standardised terminology for ethnic groups globally, the search terms applied only incorporated a selection of ethnic terms and so some ethnicities may not have been included as a result. Although this is not a meta-analysis, this systematic review involved a comprehensive search of international data and resulted in a small number of consistent findings which may help to guide future food-based dietary guidelines when considering diverse ethnic populations. 

## 5. Conclusions

This review highlighted key differences in dietary intake among frequently assessed ethnicities. Overall, Black groups globally had among the lowest fruit and vegetable intakes of groups examined. Asian groups had the highest fish intake, which contributed to their high diet quality scores, however, these groups consistently consumed minimal calcium-rich foods. The factors influencing food choice varied by ethnicity, with education and occupation levels associated with different dietary habits among groups. Minorities experienced lower proximity to shops and less food availability and, although definite trends in food availability were noted, a more global approach is needed to determine food availability among minority groups living outside the US. Future work needs also to consider extensive dietary analysis, not solely food group intake, and social and demographic confounders should be considered when examining SES influence. Intervention strategies need to be investigated further to understand which methods generate the best results and if the success of a chosen approach varies based on ethnicity.

## Figures and Tables

**Table 1:** Mean daily servings of food groups across different ethnic groups.

Author, Year	Ethnicities (Country)	Fruit	Vegetables	Meat and Fish	Dairy	Snacks/Fast Food	Cereals & Grains
Deshmukh et al., 2007	White, Black (United States)	White: 1.14 Black: 1.53 *	White: 2.27 * Black: 1.91	Meats White: 1.11, Black: 1.13 Burgers & Sandwiches White: 0.73, Black: 0.76	*Milk* White: 0.80 *, Black: 0.58 *Cheese* White: 0.43 *, Black: 0.37 *Yoghurt* White: 0.04, Black: 0.04	*Snacks & Desserts* White: 2.14 Black: 2.42 * Soft Drinks White: 1.65 * Black: 1.48	*Bread & Cereals* White: 2.26 Black: 2.30
Liu et al., 2017	White, Asian (Australia)	White: 1.76 Asian: 1.59	White: 2.34 Asian: 1.47	*Meat & Meat Products* White: 1.40, Asian: 1.10 *Meat & Meat Substitutes* White: 2.03, Asian: 1.93 *Fish* White: 0.24, Asian: 0.80	*Milk* White: 1.93 Black: 1.31	N/R	*Cereals* White: 5.41, Asian: 7.69
Patterson et al., 1995	White, Black, Hispanic (United States)	Males White: 1.10 Black: 1.14 Hispanic: 1.31 Females White: 1.29 Black: 1.34 Hispanic: 1.50	Males White: 1.94 Black: 1.71 Hispanic: 2.36 Females White: 1.94 Black: 1.74 Hispanic: 2.43	*Beef* Males White: 0.46, Black: 0.43, Hispanic: 0.57 Females White: 0.34, Black: 0.36, Hispanic: 0.44 *Chicken* Males White: 0.17, Black: 0.29, Hispanic: 0.29 Females White: 0.17, Black: 0.29, Hispanic: 0.29 *Fish* Males White: 0.07, Black: 0.14, Hispanic: 0.10 Females White: 0.07, Black: 0.14, Hispanic: 1.50	*Milk* Males White: 1.00, Black: 0.57, Hispanic: 1.00 Females White: 1.00, Black: 0.57, Hispanic: 1.00	*Snacks & Desserts* Males White: 0.93, Black: 0.79, Hispanic: 0.81 Females White: 0.74, Black: 0.73, Hispanic: 0.64 *Soft Drinks* Males White: 0.86, Black: 1.00, Hispanic: 1.00 Females White: 0.43, Black: 0.71, Hispanic: 0.57	*Cereals* Males White: 0.46, Black: 0.36, Hispanic: 0.43 Females White: 0.50, Black: 0.43, Hispanic: 0.57 *White Bread* Males White: 1.00, Black: 1.00, Hispanic: 1.00 Females White: 0.57, Black: 1.00, Hispanic: 0.71
Metcalf et al., 2008	White, Maori, Pacific Islander (PI), Asian (New Zealand)	Males White: 1.99 Maori: 1.58 PI: 2.07 Asian: 2.40 Females White: 3.14 Maori: 2.40 PI: 2.91 Asian: 2.87	Males White: 4.06 Maori: 3.36 PI: 3.67 Asian: 3.96 Females White: 5.06 Maori: 4.42 PI: 4.61 Asian: 4.20	*Red Meat* Males White: 1.06, Maori: 1.02, PI: 1.26, Asian: 0.88 Females White: 0.95, Maori: 1.02, PI: 2.91, Asian: 2.87 *Chicken* Males White: 0.16, Maori: 0.18, PI: 0.41, Asian: 0.28 Females White: 0.19, Maori: 0.20, PI: 1.00, Asian: 4.20 *Fish* Males White: 0.27, Maori: 0.39, PI: 0.60, Asian: 0.40 Females White: 0.31 Maori: 0.39, PI: 0.65, Asian: 0.46	*Milk* Males White: 2.17, Maori: 2.02, PI: 0.81, Asian: 0.85 Females White: 1.92, Maori: 1.48, PI: 1.32, Asian: 0.96 *Cheese* Males White: 0.47, Maori: 0.27, PI: 0.09, Asian: 0.15 Females White: 0.52, Maori: 0.29, PI: 0.13, Asian: 0.17	N/R	*Cereals* Males White: 0.53, Maori: 0.53, PI: 0.17, Asian:0.21 Females White: 0.58, Maori: 0.50, PI: 0.33, Asian: 0.31 *Bread* White: 0.82, Maori: 1.02, PI: 1.09, Asian: 0.64 Females White: 0.65, Maori: 0.90, PI: 1.02, Asian: 0.55
Dubowitz et al., 2008	White, Black, Hispanic (United States)	*Fruit Only* White: 1.55, Black: 1.30, Hispanic: 1.52 *Vegetables Only* White: 3.35, Black: 2.69 *, Hispanic: 3.05 *Fruit & Vegetables* White: 4.90, Black: 3.99 *, Hispanic: 4.57	N/R		N/R	N/R	N/R
Sharma et al., 2014	White, Black, Latino-US, Latino, Asian, Native Hawaiian (United States)	*Fruit* Males White: 3.10, Black: 3.20, Latino-US: 3.40, Latino: 4.20, Asian: 2.80, Native Hawaiian: 3.20 Females White: 3.30, Black: 3.70, Latino-US: 3.80, Latino: 4.90, Asian: 4.60, Native Hawaiian: 5.50 *Vegetables* Males White: 4.70, Black: 4.00, Latino-US: 4.40, Latino: 5.60, Asian: 4.60, Native Hawaiian: 5.50 Females White: 4.70, Black: 4.20, Latino-US: 4.40, Latino: 5.70, Asian: 4.70, Native Hawaiian: 5.90	N/R		N/R	N/R	N/R

N/R = not reported, * significant difference between groups (≤0.05).

**Table 2:** Mean daily macronutrient intakes across different ethnic groups.

Author, Year	Ethnicities (Country)	Energy (kcal/Day)	Protein (g/Day)	Carbohydrate (g/Day)	Fat (g/Day)	Saturated Fat (g/Day)	Fibre (g/Day)	Sucrose (g/Day)
Alonge et al., 2011	Nigerian, Mexican, Chinese (United States)	Nigerian: 1999.30 Mexican: 1833.40 Chinese: 1592.30 *	N/R	Nigerian: 331.20 Mexican: 284.50 Chinese: 210.80	N/R	N/R	Nigerian: 16.80 Mexican: 19.40 Chinese: 12.00	Nigerian: 147.60 Mexican: 130.10 Chinese: 62.20
Baroudi et al., 2009	Arab, Berbe (Tunisia)	Arab: 2044.00 Berbe: 2027.00	Arab: 55.00 Berbe: 60.00	Arab: 248.00 Berbe: 242.00	Arab: 24.00 Berbe: 27.00	Arab: 17.70 Berbe: 18.20	Arab: 24.00 Berbe: 27.00	Arab: 33.00 Berbe: 23.00 *
Hansen et al., 2011	Luo, Kamba, Maasai (Kenya)	Males Luo: 2055.45 Kamba: 1386.23 Maasai: 1601.33 Females Luo: 2509.56 Kamba: 1720.84 Maasai: 2007.65	Males Luo: 79.00 Kamba: 49.60 Maasai: 71.30 Females Luo: 63.30 Kamba: 38.50 Maasai: 58.50	Males Luo: 430.00 * Kamba: 300.00 Maasai: 273.00 Females Luo: 366.00 Kamba: 250.00 Maasai: 240.00	Males Luo: 48.90 Kamba: 33.90 * Maasai: 68.20 Females Luo: 34.30 Kamba: 22.80 Maasai: 47.00	N/R	N/R	N/R
Little et al., 2020	Black, White (United States)	Black: 1839.30 White: 1893.40	Black: 67.60 White: 77.90	Black: 221.60 White: 222.40	Black: 78.40 White: 78.60	N/R	N/R	N/R
Liu et al., 2017	White, Asian (Australia)	White: 1405.20 Asian: 1272.00	White: 72.90 Asian: 65.90 *	White: 147.80 Asian: 139.50 *	White: 58.00 Asian: 48.90 *	N/R	N/R	N/R
Metcalf et al., 2008	White, Maori, Pacific Islander (PI), Asian (New Zealand)	N/R	Males White: 91.00 Maori: 101.00 * PI: 116.00 * Asian: 102.00 Females White: 81.00 Maori: 90.00 * PI: 108.00 * Asian: 95.00	Males White: 284.00 Maori: 300.00 PI: 314.00 * Asian: 263.00 Females White: 257.00 Maori: 282.00 * PI: 311.00 * Asian: 255.00	Males White: 89.00 Maori: 99.00 PI: 105.00 * Asian: 81.00 * Females White: 76.00 Maori: 89.00 * PI: 93.00 * Asian: 74.00	Males White: 34.00 Maori: 38.00 PI: 42.00 * Asian: 32.00 Females White: 29.00 Maori: 33.00 PI: 36.00 * Asian: 27.00 *	Males White: 26.00 Maori: 24.00 PI: 26.00 Asian: 21.00 * Females White: 26.00 Maori: 26.00 PI: 28.00 Asian: 22.00 *	Males White: 60.00 Maori: 61.00 PI: 66.00 Asian: 49.00 * Females White: 58.00 Maori: 58.00 PI: 63.00 Asian: 44.00 *
Thompson et al., 2020	Black, White (United States)	Black: 2345.70 White: 2486.80	Black: 88.80 White: 96.80	Black: 277.90 White: 290.60	Black: 88.10 White: 95.10	Black: 27.80 White: 31.30	Black: 14.80 White: 18.80	Black: 130.00 White: 129.70

* significant difference between groups (≤0.05).

**Table 3:** Summary of diet quality scores among different ethnic groups.

Author, Year	Ethnicities (Country)	Diet Quality Index	Diet Quality Score	Diet Quality Drivers
Gallegos et al., 2020	Southeast Asian, South Asian, Middle East, African, Pacific Islander (Australia)	Eating Behaviour Score Scored out of 9	Southeast Asian: 5.40 South Asian: 5.20 Middle East: 3.40 African: 3.90 Pacific Islander: 4.00	Over three-quarters of Southeast Asians consumed two or more servings of fruit compared to 36.70% of Africans. Africans also consumed red and processed meat and soft drinks most frequently. Middle Eastern groups had the highest frequency of salty and sweet snacks of all groups assessed.
Hunter and Linn., 1979	Black, White (United States)	Meal Rating Score Scored out of 3 (lower score = healthier diet)	Black: 2.41 * White: 1.77	Both Black males and females had significantly higher meal rating scores than their White counterparts meaning their meal rating, protein and fatty meat intake is not as in line with recommendations as White males and females. Males of both groups had significantly poorer meal rating scores than females.
Little et al., 2020	Black, White females only (United States)	Healthy Eating Index-2010 Scored out of 100	Black: 50.00 White: 52.80	No difference in overall HEI scores or components of HEI score. Greens and beans, wholegrains and seafood and plant protein intakes were low for both groups (all < 2/5).
Nicolaou et al., 2006	Dutch, South Asian Surinamese, African Surinamese (The Netherlands)	Diet Quality Indicator Score Scored out of 7	Dutch: 3.67 * South Asian Surinamese: 4.50 African Surinamese: 4.14	Dutch groups had significantly lower diet quality scores due to significantly higher red meat and significantly lower fish and vegetable intake than other groups. Less than one third of Dutch and African Surinamese males met fruit recommendations.
Thompson et al., 2020	Black, White–males only (United States)	Healthy Eating Index-2010 Scored out of 100	Black: 46.10 White: 49.40	No significant difference in HEI scores. However, Black males scored significantly lower for vegetables, dairy, seafood and plant protein.
Yau et al., 2019	Dutch, South Asian Surinamese, African Surinamese, Moroccan, Turkish (The Netherlands)	Dutch Health Diet Index-2015 Scored out of 130	Dutch: 83.30 * South Asian Surinamese: 87.00 African Surinamese: 82.50 * Moroccan: 88.50 Turkish: 89.40	Dutch men had higher vegetable intake than men from other ethnic groups, but the lowest fruit and processed meat intake. Wholegrain, dairy and fish intakes were low among most groups. South-Asian Surinamese scored the highest for fish intake. Scores for soft drinks and fruit juice were low among African Surinamese participants.

* significant difference between groups (≤0.05).

**Table 4:** Food choice influences of ethnic groups.

Author, Year	Ethnicities (Country)	Adjusted Model	Key Findings
Baker et al., 2006	White, Black, Mixed (United States)	Racial distribution, poverty rate.	*Food Availability:* ethnicity and income: associated with location of food outlets and selection of healthy food options. In the highest tertile, 22 out of 26 supermarkets were found in Non-Hispanic White areas, none in Non-Hispanic Black areas.
Bell and Holder., 2019	White, Black (United States)	Age, class standing, parents’ education, race concordant (%).	*Environment:* Black groups significantly less likely to assume peers consume fruit and vegetables and avoid unhealthy foods daily. *Health Concerns:* associated with consuming more fruit and vegetables and less unhealthy foods. Black groups significantly less likely to report the importance of consuming fruit and vegetables and avoiding unhealthy foods daily.
Bowen et al., 2018	White, Hispanic (United States)	Age.	*SES:* high education positively associated with fruit and vegetable consumption and inversely associated with soft drink consumption (White). Positively associated with calories from fat (Hispanic). *Environment:* presence of convenience stores positively associated with fat and soft drink consumption (White). Presence of ethnic stores positively associated with fruit and vegetable consumption (Hispanic).
Dekker et al., 2015	Dutch, African Surinamese, Asian Surinamese (The Netherlands)	Age, BMI.	*SES:* higher occupation associated with higher adherence with the “vegetable” dietary pattern (all White and Surinamese females). Higher occupation levels were less likely to adhere to the “noodle and white meat” pattern (African males). Higher occupation levels were less likely to adhere to the “red meat and snacks” pattern (White groups).
Dubowitz et al., 2008	White, Black, Hispanic (United States)	Age, gender, nativity, income, education, occupation.	*Environment:* neighbourhood SES was positively associated with fruit and vegetable consumption (mostly among White groups). Nearly 50% of the difference in White and Black group fruit and vegetable intake was explained by neighbourhood SES.
Dunn et al., 2012	White, African American (United States)	N/R	*Food Availability:* White groups lower exposure to fast food outlets. Availability of fast-food outlets related to increased fast-food consumption among Black groups.
Kells et al., 2015	White, Black (United States)	Age, ethnicity, sex, region.	*SES:* association between income and education and adherence to “alcohol/salads”, “plant-based” and “sweets/fats” dietary patterns differed significantly by group. *Environment:* association between community SES and adherence to convenience patterns.
Morland and Filomena. 2007	White, Black, Hispanic (United States)	Population density, median, house value.	*Food Availability:* in NHW areas (64%), racially mixed (31%) and NHB areas (5%). Of fruit and vegetable options assessed, 15% were not available in NHB area stores.
Nicolaou et al., 2006	Dutch, South Asian Surinamese, African Surinamese (The Netherlands)	Age, marital status.	*SES:* high education associated with higher diet score and healthier eating habits (NHW). Increase in vegetable and/or fruit consumption (Surinamese females) and breakfast consumption (Asian males). *Environment:* higher social contact with White groups resulted in change in cooking practices, increased red meat intake (Asian males) and increased fish intake (African males).
Nicolaou et al., 2009	Dutch, African Surinamese, Asian, Moroccan, Turkish (The Netherlands)	N/A	*Environment:* lifestyle changes resulted in deviation from traditional meals, irregular patterns, and increased snacking (Moroccan and Turkish) *Culture:* Islam religion influences Turkish and Moroccan groups food choices—only consume halal foods, food waste is considered bad.
Pearcey and Zhan. 2018	American, Chinese (United States)	N/R	*Food Availability:* price and convenience rated significantly higher among Americans. *Health Concerns:* natural content of food and ethical concerns significantly higher among Chinese. Both considered the food’s healthiness of similar importance.
Powell et al., 2006	White, Black, Asian, Other (United States)	Population size, urbanization, region.	*Environment:* low SES neighbourhoods significantly fewer chain supermarkets available which stock more food variety and healthy options. *Food Availability:* White groups had 50% more chain supermarkets than Black groups. Hispanic areas had significantly fewer convenience stores than all other groups.
Rezazadeh et al., 2015	Turkish, Kurdish (Iran)	Energy, BMI.	*SES:* high education associated with higher adherence to “fruit and vegetable” dietary patten (Kurdish). Low occupation and income associated with higher adherence to “refined grains” dietary pattern (both).
Tovar et al., 2013	Brazilian, Latino, Other (United States)	N/A	*Food Availability:* greater food diversity available than home countries. US food prices were more expensive, especially for fresh/healthy produce (Latino). *Environment:* groups reported higher stress levels and low support in US. Time was a barrier to preparing traditional/healthy meals, eating as a family and a facilitator for fast food consumption. *Health Concerns:* all groups believed traditional foods were healthier and contained less preservatives; however, food safety concerns exist.
Wang and Chen. 2011	White, Black, Hispanic, Asian (United States)	Survey year, sex, age, education, income, region	*SES:* Higher education associated with higher HEI scores (White). SES accounted for one third of the difference between White and Black HEI scores. *Food Availability:* food price and convenience significantly less influential to White than other groups. *Health Concerns:* knowledge/awareness influenced food choice of White groups most who reported better knowledge of nutrition and health risks. This was positively associated with HEI scores.
Wang et al., 2016	White, Black, Hispanic, Asian (United States)	Age, sex, nativity, education, income.	*SES:* higher education and income level associated with fruit and vegetable intake. *Food Availability*: fresh produce availability associated with fruit and vegetable intake among White groups.
Wang et al., 2015	White, Black, Asian/Pacific Islander, Latino (United States)	N/R	*SES:* those of higher education (college graduates) and of higher income were more likely to consume fruit and vegetables daily.
Yau et al., 2019	Dutch, South Asian Surinamese, African Surinamese, Turkish, Moroccan (The Netherlands)	Age, marital status, household number, smoking status, physical activity, energy, BMI.	*SES:* low education associated with lower diet quality scores (all NHW, Asian males, African females). Low occupation associated with lower diet quality score (all NHW and Moroccans and Surinamese females).
Yeh et al., 2008	White, Black, Hispanic (United States)	N/A	*Environment:* children’s dislike of vegetables leads to food waste, not cost effective, therefore not bought (White, Black). Church community helps encourage more healthy cooking methods/ideas. *Food Availability:* lack of larger grocery stores nearby, local shops do not stock fresh produce or traditionally familiar products (Black, Hispanic). *Health Concerns:* importance of including fruit and vegetables for reducing disease risk (all groups).

N/R = not reported, N/A = not applicable (focus groups).

## Data Availability

No new data were created or analyzed in this study. Data sharing is not applicable to this article.

## Supplementary Materials

The following supporting information can be downloaded at: https://www.mdpi.com/article/10.3390/nu14050941/s1, Table S1: PICO search strategy; Table S2: Summary of studies examining dietary habits across different ethnic groups; Table S3: Summary of studies examining influencing of food choice between different ethnic groups; Table S4: Percentage consumers of food groups; Table S5: Narrative summary of dietary patterns among different ethnic groups.
