# Supplementary material for: A Comparison of Dietary Patterns and Factors Influencing Food Choice among Ethnic Groups Living in One Locality: A Systematic Review
Source: Nutrients. 2022 Feb 23;14(5):941. doi: 10.3390/nu14050941 (PMC8912306; doi:10.3390/nu14050941)
Supplement: Supplementary file 1 [file nutrients-14-00941-s001.zip › nutrients-1605654-supplementary.pdf]

Table S1. PICO Search Strategy.

**Population**	**Minority ethnic groups including:**<br>**Asian, Black, Caucasian, African, Hispanic, Latino, Eastern European**
**Interventio n**	Measurement of:<br>Diet (must assess dietary patterns, diet quality or food group intakes)<br>AND/OR<br>Determinants of food choice
**Compariso n**	Dietary comparison of at least two population groups, one of which is an ethnic group, living in one country/region<br>AND/OR<br>Comparison of factors influencing food choice of different population groups, living in one country/region
**Outcomes**	Dietary analysis among ethnic groups<br>AND/OR<br>Analysis of food choice influences of ethnic groups (including qualitative analysis)
**Study Design**	Any primary research studies such as cross-sectional<br>Exclude opinion or commentary pieces or reviews

Table S2. Summary of studies examining dietary habits across different ethnic groups.

Author, Year	Study Design	Assessment Method and Variable Assessed	Sample Size and Participant Demographics	Country, Ethnic Groups (% of total)
Abu-Saad et al., 2012	Cross-sectional	FFQ, Dietary Patters	n=1,104, 50% female, age: 25-74yrs	Israel; Arab: n=551 (50), Jewish: n=553 (50)
Adebayo et al., 2017	Cross-sectional	FFQ, Food Group	n=1,372, 54% female, age: 18-64yrs	Finland; Russian: n=527 (38), Kurdish: n=508 (37), Somali: n=337 (25)
Ahmad et al., 2016	Cross-sectional	24HDR, Questionnaire<br>Food Group, Meal/Dietary Patterns	n=2,675, 56% female, age: 18+yrs, x̄: 43.4yrs	Malaysia; Malays: n=2,058 (77), Indian: n=223 (8), Chinese: n=394 (15)
Alonge et al., 2011	Cross-sectional	24HDR, Food Group	n=213. 62% female, age: 18-93yrs, x̄: 37yrs	United States; Mexican: n=66 (31), Nigerian: n=95 (45), Chinese: n=52 (24)
Amougou et al., 2021*	Cross-sectional	24HDR, FFQ<br>Food Group, Meal Patterns	n=728, 49% female, age: 18-75yrs	Cameroon; Beti: n=349 (48), Bamileke: n=378 (52)
Baroudi et al., 2009	Cross-sectional	24HDR, FFQ<br>Meal/Dietary Patterns, Nutrients	n=94, 60% female, age: 32-64yrs	Tunisia; Berbe: n=47 (50), Arab: n=47 (50)
Beasley et al., 2020	Cross-sectional	24HDR, Nutrients	n=15,697, 51% female, age: 18+yrs	United States; White: n=10,203 (65), Black: n=1,805 (11.5), Hispanic: n=2,354 (15), Asian: n=848 (5.4), Other: n=487 (3.1)
Bell and Holder, 2019*	Cross-sectional	FFQ, Food Group	n=1,278, x̄: 22.7yrs	United States; White: n=985 (77), Black: n=293 (23)
Brenner and Kreiger, 2011	Cross-sectional	FFQ, Food Group, Meal/ Dietary Patterns, Nutrients	n=1,153, 88% female, age: 20-29yrs	Canada; White: n=560 (49), South Asian: n=119 (10), Asian: n=390 (34), Other: n=84 (7)
Bowen et al., 2018*	Cross-sectional	24HDR, Food Group	n=1,002,100% female,age: 30-50yrs	United States; White: n=493 (49), Hispanic: n=509 (51)
Costa et al., 2018*	Cross-sectional	FFQ, Food Group	n=17,410, 56% female, age: 20+yrs	Portugal; Native: n=16,123 (93), Immigrant: n=1,285 (7)
Dekker et al., 2015*	Cross-sectional	FFQ<br>Dietary Patterns, Nutrients	n=2,463, age: 18-70yrs, x̄: 49yrs	Netherlands; Dutch: n=1,254 (51), South Asian Surinamese: n=425 (17), African Surinamese: n=784 (32)
Deshmukh-Taskar et al., 2007*	Cross-sectional	FFQ, Food Group	n=1,266, 61% female, age: 20-38yrs	United States; White: n=942 (74), Black: n=324 (26)
Dubowitz et al., 2008*	Cross-sectional	24HDR, Food Group	n=13,281, 52% female, x̄: 44.57yrs	United States; White: n=5,036 (74), Black: n=3,819 (11.7), Hispanic: n=3,827 (5.5)
Gallegos et al., 2020	Cross-sectional	FFQ, Food Group	n=700, 69% female, age: 18+yrs	Australia; Southeast Asia: n=126 (18), South Asia: n=140 (20), Middle East: n=161 (23), African: n=112 (16), Pacific Islander: n=161 (23)
Hansen et al., 2011	Cross-sectional	24HDR, Food Group, Dietary Patterns, Nutrients	n=1,163, 61% female, age: 18-68yrs x̄: 38.6yrs	Kenya; Luo: n=403 (35), Kamba: n=398 (34), Maasai: n=362 (31)
Hunter and Linn., 1979	Cross-sectional	Dietary History, Questionnaire<br>Food Group, Dietary Patterns	n=182, 50% female, x̄: 72.5yrs	United States; White: n=88 (48), Black: n=94 (52)
Kell et al., 2015*	Cross-sectional	FFQ<br>Food Group, Dietary Patterns	n=17,062, 54% female, age: 45+yrs x̄: 64.7yrs	United States; White n=11,151 (65.4), Black n=5,911 (34.6)
Little et al., 2020	Cross-sectional	24HDR<br>Food Group, Diet Quality	n=89, 100% female, age: 20-76yrs x̄: 39.7yrs	United States; Black: n=50 (56), White: n= 39 (44)
Liu et al., 2017	Longitudinal	FFQ<br>Food Group, Nutrients	n=6,461, 100% female, age: in 60's	Australia; White: n=6,330 (98), Asian: n=131 (2)

Metcalf et al., 2008	Cross-sectional	FFQ<br>Food Group, Nutrients	n=4,007, 52.2% female, age: 35-74yrs	New Zealand; White: n=1,742 (43.5), Maori: n=1,003 (25), Pacific Islander: n=986 (24.6), Asian: n=276 (6.9)
Nicolaou et al., 2006*	Cross-sectional	FFQ<br>Food Group, Dietary Patterns	n=1,518, age: 35-60yrs	Netherlands; Dutch: n=552 (36), South Asian: n=307 (20), Afro-Caribbean: n=670 (43)
Pareo-Tubbeh et al., 1999	Cross-sectional	FFQ<br>Food Group, Nutrients	n=735, 47% female, age: 65-96yrs	United States; White: n=405 (55), Hispanic: n=330 (45)
Park et al., 2020	Longitudinal	FFQ, Diet Quality	n=63,255, 57% female, age: 45-75yrs	United States; Black: n=6,705 (10.6), White: n= 17,838 (28.2), Latino: n=12,145 (19.2), Native Hawaiian: 4,807 (7.6), Asian: n=21,760 (34.4)
Patterson et al., 1995	Cross-sectional	FFQ, Food Group	n=20,143, 58% female, age: 18-99yrs	United States; White: n=16,065 (80), Black: n=2,748 (14), Hispanic: n=1,330 (6)
Petrenya et al., 2019*	Cross-sectional	FFQ<br>Food Group, Dietary Patterns	n=4,504, 54% female, age: 40-69yrs	Norway; Sami: n=1,139 (25), Non-Sami: n=2,743, (61), Multi-Ethnic Sami: n=622 (14)
Rezazadeh et al., 2015*	Cross-sectional	FFQ, Food Group, Dietary Patterns, Nutrients	n=723, 59% female, age: 20-64yrs	Iran; Turkish: n=445 (62), Kurdish: n=278 (38)
Sharma et al., 2013	Cross-sectional	FFQ<br>Food Group, Nutrients	n=186,916, 54% female, age: 45-75yrs	United States; White: n=47,236 (25), Black: n=31,852 (17), Latino: n=42,951 (23), Asian: n=51,248 (27), Native Hawaiian: n=13,629 (7)
Sharma et al., 2013	Cross-sectional	FFQ<br>Food Group, Nutrients	n=186,916, 54% female, age: 45-75yrs	United States; White: n=47,236 (25), Black: n=31,852 (17), Latino: n=42,951 (23), Asian: n=51,248 (27), Native Hawaiian: n=13,629 (7)
Sharma et al., 2014	Cross-sectional	FFQ<br>Food Group, Nutrients	n=186,916, 54% female, age: 45-75yrs	United States; White: n=47,236 (25), Black: n=31,852 (17), Latino: n=42,951 (23), Asian: n=51,248 (27), Native Hawaiian: n=13,629 (7)
Siega-Riz et al., 2000	Cross-sectional	24HDR, Meal/ Dietary Patterns	n=15,641, age: 18-65yrs	United States; White, Black, Hispanic
Simmons and Williams. 1997	Cross-sectional	Questionnaire<br>Food Group, Dietary Patterns	n=6,894, 47% female, age: 20+yrs	United Kingdom; White: n=3,425 (50), Asian: n=3,469 (50)
Sorkin and Billimek et al., 2012	Cross-sectional	FFQ, Survey<br>Food Group	n=26,721, 45% female, age: 18+yrs	United States; White: n=19,264 (72), Black: n=1,749 (6.5), Asian/Pacific Islander n=1,616 (6), Latino n=4,092, (15)
Steyn et al., 2011	Cross-sectional	24HDR, Questionnaire<br>Food Group	n=3,287, age: 16+yrs	South Africa; White: n=353 (11), Black: n=1936 (59), Mixed: n=604 (18), Asian: n=388 (12)
Thompson et al., 2020	Cross-sectional	24HDR<br>Nutrients, Diet Quality	n=5050, 100% male, age: 20+yrs	United States; Black: n=1,566 (31.3), White: n=3,484 (68.7)
Tichenor and Conrad, 2014	Cross-sectional	FFQ, Survey<br>Food Group	n=275,86, age: 18+yrs	United States; White, Black, Hispanic, Other
Wang et al., 2016*	Cross-sectional	FFQ, Food Group	n=36,302, 58% female, age: 18+yrs	United States; White: n=24,686 (68), Black: n=1,815 (5), Hispanic: n= 6,171 (17), Asian: n=3,630 (10)
Yau et al., 2019*	Cross-sectional	FFQ<br>Food Group, Diet Quality	n=4,632, 59% female, age: 18-70yrs	Netherlands; Dutch: n=1,452 (31), South Asian Surinamese: n=971 (21), African Surinamese: n=944 (20), Moroccan: n=687 (15), Turkish: n=578 (13)

*= examined both dietary intake and food choice influences among ethnic groups, 24HDR=24-hour dietary recall, FFQ=Food Frequency Questionnaire, yrs= years, $\bar{x}$=mean age of cohort.

Table S3. Summary of studies examining influencing of food choice between different ethnic groups.

Author, Year	Study Design	Assessment Method and Variable Assessed	Sample Size and Participant Demographics	Country, Ethnic Groups (% of total)
Amougou et al., 2021*	Cross-sectional	International Wealth Index, SES	n=728, 49% female, age 18-75 yrs	Cameroon; Beti: n=349 (48), Bamileke: n=378 (52)
Baker et al., 2006	Cross-sectional	Supermarket Fast-Food Restaurant Audits, Census Analysis<br>SES, Food Availability	N/A	United States; White, Black, Mixed
Bell and Holder, 2019*	Cross-sectional	SES Questionnaire<br>SES, Social Influences	n=1,278, x̄: 22.7 yrs	United States; White: n=985 (77), Black: n=293 (23)
Bowen et al., 2018*	Cross-sectional	Questionnaire<br>SES, Food Availability	n=1,002, 100% female, age: 30-50 yrs	United States; White n=493 (49), Hispanic: n=509 (51)
Costa et al., 2018*	Cross-sectional	Lifestyle Questionnaire<br>SES, Lifestyle	n=17,410, 56% female, age: 20+ years	Portugal; Native: n=16,123 (93), Immigrant: n=1,285 (7)
Dekker et al., 2015*	Cohort	SES Questionnaire, SES	n=2,463, age: 18-70yrs, x̄: 49 years	Netherlands; Dutch: n=1,254 (51), South Asian Surinamese: n=425, (17), African Surinamese: n=784 (32)
Deshmukh-Taskar et al., 2007*	Cross-sectional	SES Questionnaire, SES	n=1,266, 61% female, age: 20-38 yrs	United States; White: n=942 (74), Black: n=324 (26)
Dubowitz et al., 2008*	Cross-sectional	Census Analysis, SES	n=13,281, 52%, x̄: 44.57 yrs	United States; White: n=5,036 (74), Black: n=3,819 (11.7), Hispanic: n=3,827 (5.5)
Dunn et al., 2012	Cross-sectional	SES Questionnaire<br>Food Availability	N/A	United States; White, Black
Kell et al., 2015*	Cross-sectional	SES Questionnaire, SES	n=17,062, 54% females, age: 45+ yrs	United States; White: n=11,151 (65.4), Black: n=5,911 (34.6)
Morland and Filomena, 2007	Cross-sectional	Supermarket Survey, Census Analysis, Food Availability	N/A	United States; White, Black, Hispanic
Nicolaou et al., 2006*	Cross-sectional	Questionnaire, SES	n=1,518, age: 35-60 yrs	Netherlands; Dutch: n=552 (36), South Asian: n=307 (20), Afro-Caribbean: n=670 (43)
Nicolaou et al.,2009	Qualitative	Food Groups, Cultural Traditions	n=79, 56% female, age: 20-40 yrs	Netherlands; Turkish n=49 (62), Moroccan: n=30 (38)
Pearcey and Zhan, 2018	Cross-sectional	Food Choice Questionnaire<br>SES, Food Availability, Cultural Traditions	n=661, 72% female, age: 18-52 yrs, x̄: 20.8 years	United States; White: n=328 (50), Black n=333 (50)
Petrenya et al., 2019*	Cross-sectional	SES Questionnaire<br>SES, Cultural Traditions	n=4,504, 54% female, age: 40-69 yrs	Norway; Sami: n=1,139 (25), Non-Sami: n=2,743 (61), Multi-Ethnic Sami: n=622 (14)
Powell et al., 2006	Cross-sectional	Zip Code, Census Analysis<br>SES, Food Availability	N/A	United States; White, Black, Asian, Other
Rezazadeh et al., 2015*	Cross-sectional	SES Questionnaire, SES	n=723, 59% female, age: 20-64 yrs	Iran; Turkish: n=445 (62), Kurdish: n=278 (38)
Steyn et al., 2011	Cross-sectional	Questionnaire, SES	n=3,287, age: 16+ yrs	South Africa; White: n=353 (11), Black: n=1936 (59), Mixed: n=604 (18), Asian: n=388 (12)

Tovar et al., 2013	Qualitative	Focus Groups<br>Food Availability,Cultural Traditions	n=25, 100% female, age: 20-55 yrs	United States; Brazilian, Latino, Other
Wang and Chen, 2011	Cross-sectional	Health Questionnaire, SES	n=4,356, 49% female, age: 20-65 yrs	United States; White, Black, Hispanic, Asian
Wang et al., 2016*	Cross-sectional	Questionnaire<br>SES, Food Availability	n=36,302, 58% female, age: 18+ yrs	United States; White: n=24,686 (68), Black: n=1,815 (5), Hispanic: n= 6,171 (17), Asian: n=3,630 (10)
Wang et al., 2015	Cross-sectional	Survey, Census Analysis, SES	n=2,669, 100% female, 20+ yrs	United States; White: n=874 (33), Black: n=318 (12), Asian/Pacific Islander: n=269 (10), Latino-US born: n=437 (16), Latino: n=771 (29)
Yau et al., 2019*	Cross-sectional	Questionnaire, SES	n=4,632, 59% female: age: 18-70 yrs	Netherlands; Dutch: n=1,452 (31), South Asian Surinamese: n=971 (21), African Surinamese: n=944 (20), Moroccan: n=687 (15), Turkish: n=578 (13)
Yeh et al., 2008	Qualitative	Structured Focus Groups<br>SES, Food Availability, Cultural Traditions	n=147, age: 18 yrs	United States; White, Black, Hispanic

*= examined both dietary intake and food choice influences among ethnic groups, SES=socioeconomics, FFQ=Food Frequency Questionnaire, 24HDR=24-hour dietary recall, yrs= years, $\bar{x}$=mean age in years.

Table S4. Percentage consumers of food groups.

Author, Year	Ethnic Group (Country)	Fruit		Vegetables		Meat		Fish		Snacks/Fast Food
Adebayo et al., 2017	Russian, Kurdish, Somali (Finland)	*Daily Consumption*<br>Males<br>Russian: 48%<br>Kurdish: 58%<br>Somalian: 0%	*Daily Consumption*<br>Females<br>Russian: 66%<br>Kurdish: 68%<br>Somalian:1%	*Daily Consumption*<br>Males<br>Russian: 55%<br>Kurdish: 38%<br>Somalian: 0%	*Daily Consumption*<br>Females<br>Russian: 62%<br>Kurdish: 52%<br>Somalian: 2%	N/R		*≥ 2 times weekly*<br>Males<br>Russian: 43%<br>Kurdish: 21%<br>Somalian: 41%	*≥ 2 times weekly*<br>Females<br>Russian: 43%<br>Kurdish: 17%<br>Somalian: 34%	N/R
Bell and Holder, 2019	White, Black (United States)	*Daily Consumption*<br>White: 43%<br>Black: 26%*		*Daily Consumption*<br>White: 48%<br>Black: 30%*		N/R		N/R		*Daily Consumption*<br>White: 62%*<br>Black: 73%
Nicolaou et al., 2006	Dutch, South Asian, Afro-Caribbean (Netherlands)	N/R		*Daily Consumption*<br>Males<br>Dutch: 54%<br>South Asian:80%<br>Afro-Caribbean: 62%	*Daily Consumption*<br>Females<br>Dutch: 61%<br>South Asian: 61%<br>Afro-Caribbean: 60%	*≤ 2 times weekly*<br>Males<br>Dutch: 23%<br>South Asian: 73%<br>Afro-Caribbean: 53%	*≤ 2 times weekly*<br>Females<br>Dutch: 34%<br>South Asian: 79%<br>Afro-Caribbean: 64%	*≥1 times weekly*<br>Males<br>Dutch: 52%<br>South Asian: 77%<br>Afro-Caribbean: 73%	*≥1 times weekly*<br>Females<br>Dutch: 51%<br>South Asian: 85%<br>Afro-Caribbean: 68%	N/R
Tichenor and Conrad, 2014	White, Black, Hispanic, Other (United States)	*Monthly Consumption*<br>White: 96%<br>Hispanic: 96%<br>Black: 96%<br>Other: 96%		*Monthly Consumption*<br>White: 43%<br>Black: 29%<br>Hispanic: 46%<br>Other: 47%		N/R		N/R		N/R
Wang et al., 2015	White, Black, Asian/Pacific Islander, Latino (United States)	*Daily F&V Consumption, Every Second Day, ≤2 times weekly*<br>White: 51%, 14%, 5%<br>Black: 35%, 21%, 10%<br>Asian/Pacific Islander: 39%, 19%, 5%<br>Latino: 32%, 25%, 14%				N/R		N/R		N/R

N/R=not reported, *=significant difference between groups ($p≤0.05$), F&V= fruit and vegetables

Table S5. Narrative summary of dietary patterns among different ethnic groups.

Author, Year	Ethnic Group (Country)	Dietary Patterns	Key Findings
Abu-Saad et al., 2012	Arab, Jewish (Iran)	N= 4 dietary patterns:<br>Ethnic, Healthy, Fish & Meat Dishes, Fast Food	Arabs significantly more likely to be high consumers of Ethnic.<br>Jewish significantly more likely to follow healthy.<br>Minimal difference between groups in Fish & Meat Dishes and Fast Food.
Brenner and Kreiger, 2011	White, South Asian, Asian, Other (Canada)	N= 3 dietary patterns:<br>Prudent, Eastern, Western.	Prudent: White groups had significantly higher Prudent pattern scores than all other groups.<br>Eastern: Asian group had significantly higher Eastern pattern scores than all other groups.
Dekker et al., 2015	Dutch, African Surinamese, Asian Surinamese (Netherlands)	N=3 dietary patterns:<br>Noodle/Rice & White Meat,<br>Red Meat, Sweets & Snacks, Vegetable, Nuts & Fruit.	African and Asian Surinamese groups more likely to adhere Noodle/Rice and White Meat pattern than Dutch.<br>Dutch were significantly more likely to adhere to Red Meat, Sweets and Snacks than all other groups.<br>Dutch were significantly more likely to adhere to Vegetable, Nuts and Fruit: than all other groups.<br>Ethnic differences were most significant between males.
Petrenya et al., 2019	Sami Non-Sami, Multi-Ethnic Sami (Norway)	N=6 dietary patterns:<br>Processed Meat/ Westernised, Fish/ Traditional, Fruit/Vegetables, Reindeer/Traditional, Bread and Sandwich spreads, Sweets & Baked Goods.	Processed Meat/ Westernised: Inland Sami had lowest scores.<br>Fish/ Traditional: Coastal Multi-Ethnic Sami had the highest scores, Inland Sami and Mult-Ethnic Sami had the lowest.<br>Bread and Sandwich spreads: positively associated with being inland Sami.<br>Sweets & Baked Goods: no ethnic differences found.
Rezazadeh et al., 2015	Turkish, Kurdish (Iran)	N=3 dietary patterns:<br>Traditional High SES, Traditional Low SES, Transitional.	Traditional High SES: significantly higher percentage of Turkish than Kurdish were within the highest tertile.<br>Traditional Low SES: significantly higher percentage of Kurdish than Turkish was in the highest tertile, while significantly more Turkish were in the lowest tertile.<br>Transitional: No significance difference between the two ethnic groups.
